# Fractionated stereotactic radiosurgery for patients with skull base metastases from systemic cancer involving the anterior visual pathway

**DOI:** 10.1186/1748-717X-9-110

**Published:** 2014-05-08

**Authors:** Giuseppe Minniti, Vincenzo Esposito, Enrico Clarke, Claudia Scaringi, Alessandro Bozzao, Teresa Falco, Vitaliana De Sanctis, Maurizio Maurizi Enrici, Maurizio Valeriani, Mattia Falchetto Osti, Riccardo Maurizi Enrici

**Affiliations:** 1 Radiation Oncology Unit, Sant’ Andrea Hospital, University Sapienza, Via di Grottarossa 1035, 00189 Rome, Italy; 2 IRCCS Neuromed, 86077 Pozzilli, IS, Italy; 3 Neuroradiology Unit, Sant’ Andrea Hospital, University Sapienza, 00189 Rome, Italy; 4 Ophthalmology Unit, S. Andrea Hospital, University Sapienza, 00189 Rome, Italy

**Keywords:** Stereotactic radiosurgery, Skull base metastases, Hypofractionated stereotactic radiosurgery, Radiation-induced optic neuropathy

## Abstract

**Background:**

To analyze the tumor control, survival outcomes, and toxicity after stereotactic radiosurgery (SRS) for skull base metastases from systemic cancer involving the anterior visual pathway.

**Patients and methods:**

We have analyzed 34 patients (23 females and 11 males, median age 59 years) who underwent multi-fraction SRS for a skull base metastasis compressing or in close proximity of optic nerves and chiasm. All metastases were treated with frameless LINAC-based multi-fraction SRS in 5 daily fractions of 5 Gy each. Local control, distant failure, and overall survival were estimated using the Kaplan-Meier method calculated from the time of SRS. Prognostic variables were assessed using log-rank and Cox regression analyses.

**Results:**

At a median follow-up of 13 months (range, 2–36.5 months), twenty-five patients had died and 9 were alive. The 1-year and 2-year local control rates were 89% and 72%, and respective actuarial survival rates were 63% and 30%. Four patients recurred with a median time to progression of 12 months (range, 6–27 months), and 17 patients had new brain metastases at distant brain sites. The 1-year and 2-year distant failure rates were 50% and 77%, respectively. On multivariate analysis, a Karnofsky performance status (KPS) >70 and the absence of extracranial metastases were prognostic factors associated with lower distant failure rates and longer survival. After multi-fraction SRS, 15 (51%) out of 29 patients had a clinical improvement of their preexisting cranial deficits. No patients developed radiation-induced optic neuropathy during the follow-up.

**Conclusions:**

Multi-fraction SRS (5 x 5 Gy) is a safe treatment option associated with good local control and improved cranial nerve symptoms for patients with a skull base metastasis involving the anterior visual pathway.

## Introduction

The base of the skull is a less common site of metastases; however, they represent a clinical challenge as growing lesions in such area that compress optic nerve, chiasm, and nerves involved in the extraocular movement have been associated with serious morbidity [1]. Therapeutic options include radiotherapy, chemotherapy, and surgery. Surgical resection is reserved for a minority of well-selected patients, depending on the accessibility of the lesion and the potential morbidity of the procedure [2]. Similarly, chemotherapy may have a role only in a subset of patients with chemosensitive or hormonosensitive lesions [3]. Palliative radiotherapy with doses of 20–30 Gy in 5–10 fractions results in significant symptom improvement [4-9], although in clinical practice many physicians prefer to withhold whole brain radiation therapy (WBRT) since it may result in a decline of neurocognitive function and quality of life [10,11].

Stereotactic radiosurgery (SRS), which has been used for nearly 30 years in patients with a limited number of brain metastases, is an effective treatment associated with excellent local control without compromising survival, and potentially avoiding the risk of the detrimental neurocognitive effects of WBRT [12,13]. Few series including either skull base metastases or head and neck cancers have reported a 1-year local control of 53%-89% after single-fraction SRS [14-17]. However, single large doses may be associated with an increased risk for neurologic morbidity from radiation necrosis [18-20], and this is of concern especially for lesions larger than 2.5-3.0 cm or of any size that are in close proximity to critical structures, such as the optic apparatus or brainstem in case of metastases of the skull base. Thus, multi-fraction SRS (up to 5 fractions) has been employed in patients with brain metastases as an alternative to single-fraction SRS with the aim to reduce late radiation-induced toxicity while maintaining high local control rate. Differing from single-fraction SRS where patients have been traditionally immobilized in an invasive frame, a stereotactic mask is usually employed for multi-fraction SRS with a setup accuracy of 0–3 mm. In patients with brain metastases multi-fraction SRS at doses of 27–42 Gy in 3–5 fractions has resulted in 1-year local control of 70-90% with acceptable radiation-induced toxicity [21-25], although the efficacy and safety of this approach in patients with a skull base metastasis remains to be determined.

In the present study we reviewed our experience with SRS given in five daily fractions in patients with skull base metastases from systemic cancer involving the anterior visual pathway. Local control, neurologic outcome and risk of radiation-induced neuropathies have been evaluated.

### Patients and methods

#### Patient population

Patient data were obtained from a prospectively maintained database of patients with brain tumors treated with brain stereotactic irradiation at our institution. We identified 34 consecutive patients who received multi-fraction SRS for a sellar and/or parasellar metastasis adjacent or compressing the optic chiasm and/or optic nerves derived from different histologically confirmed systemic cancers. They represent the 2.3% of all patients with brain metastases treated with SRS between March 2005 and August 2013 at University of Rome Sapienza, Sant’Andrea Hospital. All patients were aged 18 years or older with no prior WBRT. Informed consent was obtained from all patients prior to treatment. The protocol was approved by the Institutional Review Board.

### Treatment

**Figure 1:**
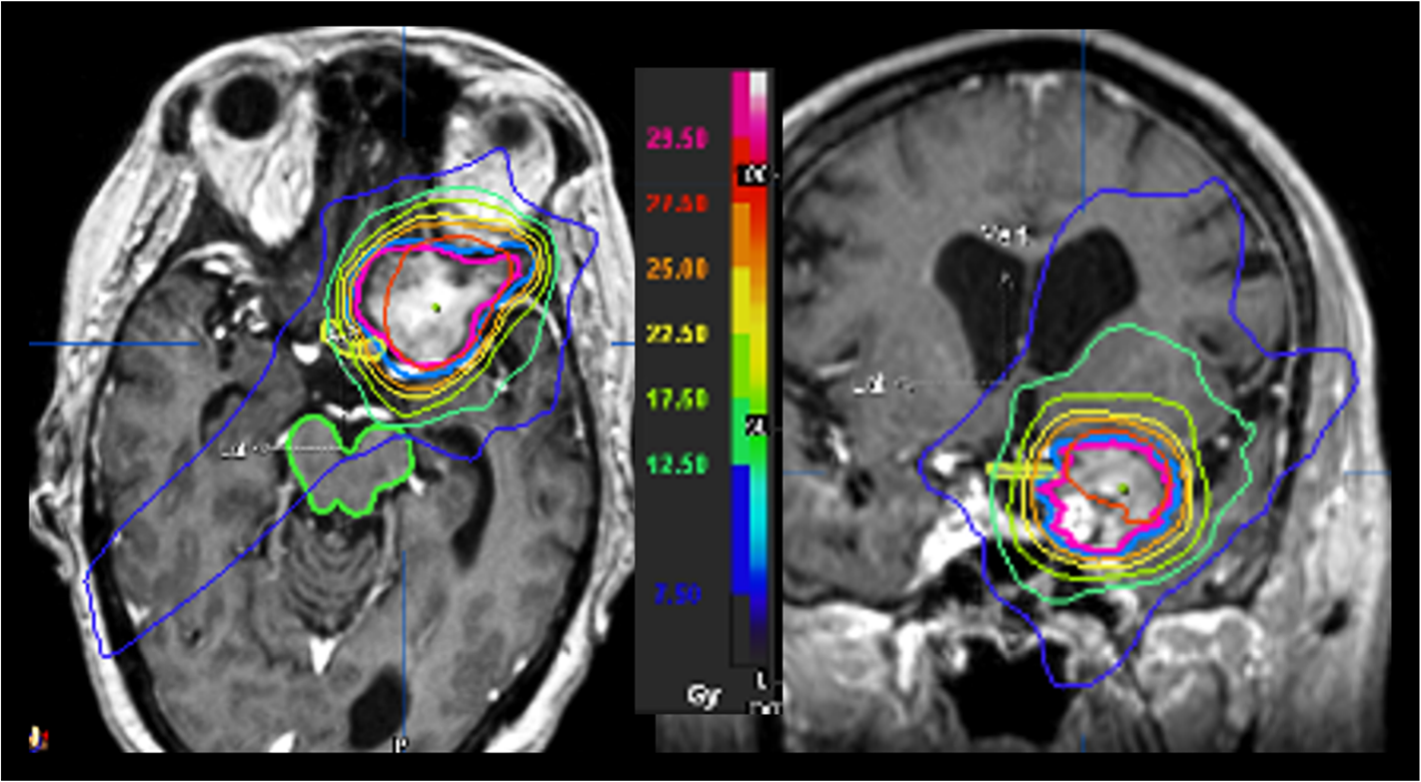
**Prescription isodose distribution in axial and coronal views of a skull base metastasis compressing the optic chiasm (contoured in yellow).** The planning target volume (contoured in blue) was created by the geometric expansion of tumour plus 1 mm. The patient was treated with a linear accelerator (LINAC) multi-fraction stereotactic radiosurgery (5 x 5 Gy) with the use of 8 noncoplanar conformal beams. The dose was prescribed to the 87% isodose line normalized to the maximum dose, with a minimum PTV coverage of 98%. The isodose curves showing dose levels delivered to surrounding tissues and adjacent critical structures are represented.

All metastases were treated with frameless LINAC-based multi-fraction SRS given in 5 daily fractions of 5 Gy. Based on the linear quadratic model 25 Gy SRS (5 x 5 Gy) is probably ’biologically equivalent’ to approximately a single-fraction SRS of 15 Gy assuming an α/β ratio of 10 Gy for the tumor, although with a potential decreased risk of damage to normal brain, including brainstem, optic nerves and chiasm (α/β ratio of 1–3 Gy). Patient immobilization was achieved by using a commercially available head mask fixation system (Brainlab AG, Feldkirchen, Germany), together with a mouth bite positioned against the upper dentition and attached to the stereotactic frame. The characteristics of the system and the technique have been previously described [26]. The target volumes were identified on the basis of the fused CT and magnetic resonance image (MRI) scans. The gross tumor volume (GTV) was delineated as a contrast-enhancing tumor demonstrated on MRI scans. The planning target volume (PTV) was generated by the geometric expansion of GTV plus 1–2 mm. Doses were prescribed to the 80-90% isodose line normalized to the maximum dose, performed to ensure coverage of at least 95% of the PTV with the prescription dose (Figure 1). Treatment volumes were achieved with 6–15 noncoplanar conformal beams by using a BrainLAB m3 micromultileaf collimator attached to a Varian Clinac 600 DBX. Patients were treated in 5 consecutive days in outpatient clinic. Dexamethasone therapy was started by the first day of treatment at doses of 4 mg PO per day and maintained for one week after the end of treatment.

### Follow-up

Patients were examined clinically before SRS and then every 2 months. At each visit, neurological status and the severity of complications were rated according to the National Cancer Institute (NCI) Common Terminology Criteria for Adverse Events v4.0 (CTCAE) central nervous system toxicity. Ophthalmologic examinations including visual acuity assessment and visual field testing were performed every 3 months. MRI was made every 2 months in the first year after treatment, and then every 3 months or as appropriate according to the neurological conditions. Complete response was defined as total radiographic disappearance of enhancing lesion, partial response as shrinkage of tumor volume >50%, and stable disease as reduction in the tumor volume ≤50%. Local progression was defined as radiographic increase in the volume of metastatic lesion on serial imaging. In patients with suspected local progression, dynamic susceptibility-weighted contrast-enhanced (DSC) perfusion MRI and 3,4-dihydroxy-6-[18]F-fluoro-l-phenylalanine ([18]F-FDOPA) PET/CT were used for differential diagnosis between recurrent tumor and radiation-induced brain necrosis. Distant intracranial progression was defined by presence of new brain metastases or leptomeningeal enhancement outside the irradiated volume. All images were reviewed by the same neuroradiologist (A.B). Tumor volumetry was performed by using the BrainLab iPlan treatment planning software and changes in tumor volume were calculated after fusion of pre-treatment and post-treatment MRI scans. Metastatic lesions were manually contoured by the same radiation oncologist (G.M) using postcontrast axial T1-weighted images with 1 mm slice thickness. For all patients who died, the cause of death (intracranial versus extracranial progression) was determined by clinical/neurological evaluation and brain/systemic radiologic studies.

Local control, distant failure, and overall survival were estimated using the Kaplan-Meier method calculated from the time of multi-fraction SRS. For univariate analysis, the log-rank test was used for categorical variables, and the Cox proportional hazards model was used for continuous variables. The multivariate Cox proportional hazards regression model was used to test the independent effect of significant prognostic factors at univariate analysis (p <0.05).

## Results

### Patient and treatment characteristics

Between March 2005 and August 2013, 34 patients who underwent multi-fraction SRS for a skull base metastasis adjacent or compressing the optic chiasm and/or optic nerves were evaluated in this study. Patients and tumor characteristics are summarized in Table 1. Twenty-three (67%) patients presented with a single skull base metastasis and 11 (33%) patients had also 1–2 brain metastases in other brain locations that were treated with SRS (8 patients) or surgery plus SRS (2 patients). At SRS, extracranial disease was absent in 13 patients. Twenty-nine had cranial nerve deficits, including optic nerve (reduced acuity in 7 and visual field defects in 9), oculomotor (n = 10), abducens (n = 9), trigeminal (n = 5), and facial (n = 2) deficits. Twenty patients received chemotherapy during the post-SRS follow-up. All patients received the planned dose 25 Gy delivered in 5 daily fractions. The median minimum PTV coverage was 97% (range, 95-100%). Median prescription isodose was 86% (82%-90%) and median conformity index was 1.61 (range 1.28-2.12). Data were reported to January 2014. At this time 25 patients had died.

**Table 1:** Summary of patient characteristics

**Parameter**	**No (%)**
***Number of patients***	34
***Sex (F/M)***	23/11
***Age (years)***	
Median	59
< 65	23
≥ 65 years	11
***Histology***	
NSCLC	12
Breast carcinoma	16
Colon carcinoma	2
Others*	4
***KPS***	
Median	70
>70	18
≤70	16
***Extracranial diseae***	
Present	21
Absent	13
***Number of metastases***	
Single	23
Multiple [2,3]	11
**Location**	
CS only	7
CS and retrorbital space	4
CS and clivus	6
CS and/or sellar/suprasellar space	14
CS and petrus bone	3
***GTV (cm***^***3***^ ***)***	
Median	8.5
Range	1.9 - 25.8
***PTV (cm***^***3***^ ***)***	
Median	12.8
Range	3.4 - 35.8
***Conformity index***	
Median	1.61
Range	1.28-2.12

KPS, Karnofsky Performance Status; GTV, Gross Target Volume; PTV, Planning Target Volume.

*Others histologies included two melanomas, one renal cell, and one prostate carcinoma.

**Figure 2:**
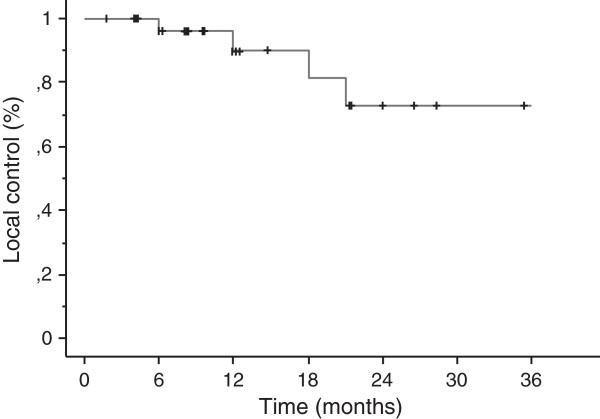
Kaplan-Meier estimate of local control.

**Figure 3:**
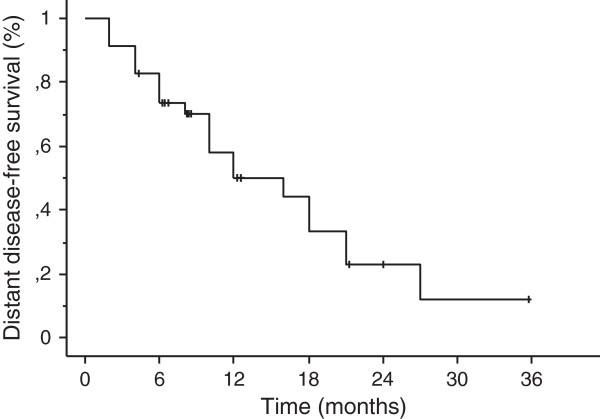
Kaplan-Meier estimate of distant disease-free survival.

**Figure 4:**
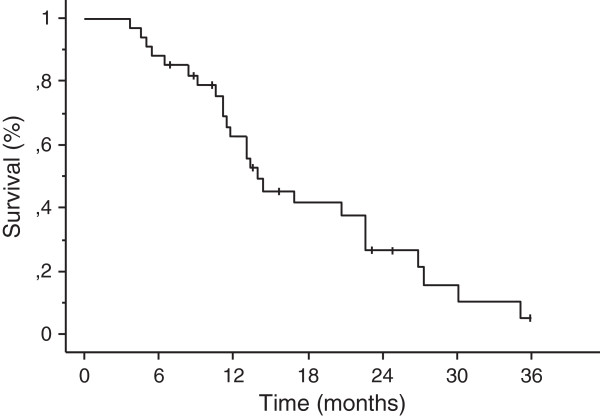
Kaplan-Meier estimate of overall survival.

With a median follow-up of 13 months (range, 2–36.5 months), crude local control was achieved in 30 cases (88%). Based on Kaplan-Meier analysis, the 1-year and 2-year local control rates were 89% and 72%, respectively (Figure 2). A complete response was achieved in 3 patients and partial response 14 patients, respectively. Four patients recurred with a median time to progression of 12 months (range, 6–27 months). Eighteen patients had new brain metastases at distant sites. The median distant metastases-free survival time was 12 months (range, 2–36 months) (Figure 3). The 1-year and 2-year actuarial incidence rates of new distant brain metastases were 50% and 77%, respectively. The median survival time was 14.5 months (95% CI, 12.0 -17.0 months), and 1-year and 2-year actuarial survival rates were 63% and 32%, respectively (Figure 4). Seventy-six percent of patients succumbed to their extracranial disease and 24% of patients died of progressive intracranial disease at either local (n = 1) and/or distant sites (n = 5).

The impact of prognostic factors on overall survival and distant metastases-free survival rates are shown in Table 2. On multivariate analysis the absence of extracranial metastases (P = 0.01; HR 0.18, 95% CI 0.07-0.64) was the only variable associated with lower distant failure rates. No factors, including histology, tumor volume and conformity index had impact on local control. The absence of extracranial metastases (p = 0.001; HR 0.1, 95% CI 0.05-0.40) and KPS >70 (p = 0.01; HR 0.30, 95% CI 0.1-0.68) were independent favorable prognostic factors for overall survival.

**Table 2:** Univariate and multivariate analysis of prognostic factors for overall survival and distant failure

**Variable**	**Median**	**Univariate**	**Multivariate analysis**	
	**Survival time (months)**	**Analysis P value**	**Hazard ratio (95% CI)**	**P value**
**Overall survival**				
Sex, male vs female	male (16.1); female (13.5)	0.1		
Age (years), < 65 vs ≥ 65	< 65 (15.0); ≥ 65 (11.5)	0.1		
KPS, ≤ 70 vs >70	≤ 70 (11.5); >70 (20.3)	0.001	0.30 (0.1-0.68)	0.01
Primary tumor, breast cancer vs others	breast cancer (13.0); others (15.5)	0.2		
Extracranial metastases, absent vs present	absent (22.5); present (11.2)	0.0001	0.10 (0.05-0.40)	0.001
Number of metastases, 1 vs > 1	1 met (15.5); >1 met (13.2)	0.3		
**Distant brain failure**				
Sex, male vs female	male (16.1); female (13.5)	0.9		
Age (years), < 65 vs ≥ 65	< 65 (12.0); ≥ 65 (10.0)	0.2		
KPS, ≤ 70 vs >70	≤ 70 (8.0); >70 (16.0)	0.03	0.36 (0.16-1.27)	0.1
Primary tumor, breast cancer vs others	breast cancer (10.0); others (14.0)	0.1		
Extracranial metastases, absent vs present	absent (18.0); present (8.0)	0.002	0.18 (0.07-0.64)	0.01
Number of metastases, 1 vs > 1	1 met (16.0); > 1 met (10.0)	0.1		

KPS, Karnofsky Performance Status.

### Neurologic function and toxicity

Cranial deficits were present in 29 (85%) out of 34 patients. After multi-fraction SRS, fifteen (51%) patients had a clinical improvement of neurological deficits. Vision acuity and/or visual fields defects improved in 7 patients, oculomotor deficits in 10 patients, trigeminal deficits in 2 patients, and facial palsy in one patient. The maximum and median doses to the optic chiasm, optic nerve, and cavernous sinus are showed in Table 3. The optic chiasm was < 2 mm away or in contact with the tumor in 10 and 8 patients, respectively. The median volume receiving > 5 Gy and the maximum dose to the optic chiasm were 0.13 cm^3^ and 5.7 Gy, respectively. No patients developed radiation-induced optic neuropathy. One patient had a transient 6^th^ nerve palsy 12 months after SRS with full recovery after a short course of corticosteroids. For this patient the maximum radiation dose was 27.5 Gy (5.5 Gy each fraction) to the cavernous sinus. Three patients experienced neurological deficits (motor deficits in 2 patients, ataxia in one patient, and speech deficits in one patient) that regressed with the use of corticosteroids. Neurological deterioration was associated with intracranial progression at distant sites in all patients. Salvage treatment consisted of further SRS (n = 10) or surgery (n = 3) in 13 patients and WBRT (30 Gy in 10 daily fractions over two weeks) in 7 patients. Median survival after WBRT was 6.3 months (range 1.8-13.5 months). No patients developed cranial deficits after salvage WBRT.

**Table 3:** Median radiation doses to cavernous sinus, optic nerves and chiasm in patients treated with multi-fraction SRS (5 × 5 Gy)

**Site**	**Median**	**Maximum**	**Organ at risk**	**Volume (cm**^**3**^**)**	**Volume (cm**^**3**^**)**
	dose	dose	volume	receiving	receiving
	(Gy)	(Gy)	(cm^3^)	a dose > 25.0 Gy	a dose > 27.5 Gy
Cavernous sinus	18.5	29	1.9 (1.44-2.52)	1.7 (1.41-2.4)	0.52 (0.2-1.9)
Optic nerve	13.5	28.5	0.93 (0.71-1.26)	0.08 (0.01-0.5)	0.026 (0.005-0.08)
Optic chiasm	5.5 (12*)	28.5	0.48 (0.33-0.71)	0.13 (0.03-0.28)*	0.022 (0.01-0.06)*

*In 18 patients receiving a total dose to the optic chiasm >25 Gy.

## Discussion

Palliative RT has been the standard treatment for skull base malignancies providing excellent relief of pain and improvement of cranial nerve dysfunction in up to 78% of patients [4-9]. More recently, SRS has been employed as a less invasive option for the treatment of skull base metastases with the aim to deliver a high dose to the target with dose sparing of critical structures such as the optic nerves and chiasm. The efficacy of single-fraction SRS for skull base metastases has been reported in few studies that include either nasopharyngeal carcinomas or skull base metastases [14-17]. Iwai et al. [15] treated 21 patients with cavernous sinus cancers, including 12 patients with metastases from systemic cancer. At a median follow-up of 13 months, the 1-year and 2-year tumor control rates were 68% and 47%, respectively, with no significant differences between nasopharyngeal carcinoma and metastases. After SRS, there was a resolution or improvement of preoperative cranial nerve deficits in 47% of patients. Kano et al. [17] reviewed retrospectively 37 patients with cancer involving the cavernous sinus treated with gamma knife SRS at the University of Pittsburgh between 1992 and 2006. At median follow-up of 9 months the progression-free survival rates after SRS were 77.6% and 26.6% at 1 and 2 years, respectively. Lower marginal doses were associated with shorter progression-free survival, as in case of tumors in close proximity to the optic pathway or larger lesions; the progression-free survival rate was 89.7% for patients receiving a marginal dose ≥ 15 Gy as compared with 35.9% for those receiving < 15 Gy. Approximately one-third of patients had improvement in their neurologic deficits, similarly to that reported in other few series [14,16].

Multi-fraction SRS represents an alternative to single-fraction SRS for relatively large brain metastases > 3 cm or adjacent to critical structures with a reported local control rates of 65-90% at 1 year [21-25]. In the current study we have evaluated 34 patients with a skull base metastasis compressing or in close proximity of the visual pathway who were treated with multi-fraction SRS (5 × 5 Gy). The 1-year and 2-year survival rates were 63% and 32%, and respective actuarial 1-year and 2-year local control rates were 89% and 69%, which are in the best range of results observed after single-fraction SRS [14-17] and fractionated stereotactic radiotherapy [27]. A tumor local control of 66-76% at 1 year has been reported following multi-fraction SRS with 5 × 5–7 Gy in patients with brain metastases [21-25]. Ernst-Stecken et al. [22] reported the treatment results of a prospective study of multi-fraction SRS (5 x 6–7 Gy) in 51 patients with 72 brain metastases. At a median follow-up of 7 months the 1-year local control was 76% and survival 60%, respectively. Using a median prescribed dose of 25 Gy with a median of 5 fractions Kwon et al. [25] observed a similar actuarial local tumor control rates of 94% and 68.2% at 6 months and 1 year, respectively, in 36 patients with 66 brain metastases. While the optimal dose and fractionation for brain metastases remain to be determined, our results indicate that multi-fraction SRS with 5 × 5 Gy is an effective treatment for skull base metastases associated with good local control and improvement of cranial deficits similar to that reported after single-fraction SRS.

The main reason for using multi-fraction SRS is the advantage of fractionation with respect to radiobiology and normal brain protection. Single-fraction SRS for skull base lesions is in fact limited by the potential toxicity of high single doses to the cranial nerves. While the reported tolerance of cranial nerves in the cavernous after single-fraction SRS sinus is of 16–18 Gy [28], several retrospective studied have indicated that the incidence of radiation-induced optic neuropathy is about 2% for doses of 8–12 Gy, and becomes >10% for doses of 12–15 Gy [28-31]. Leavitt et al. [31] have recently reviewed 222 patients treated with Gamma Knife SRS for benign tumors adjacent to the anterior visual pathway. The risk of optic neuropathy was 0 for patients receiving a maximum dose of 8–12 Gy and 10% for those receiving >12 Gy, respectively, suggesting that small portions of anterior visual pathway in the range of 0.02-0.04 cm^3^ may receive doses up to 12 Gy. In our study no optic neuropathy were observed for doses >25 Gy to less than one-third of optic chiasm and > 27.5 Gy to a small volume of 0.01-0.06 cm^3^. These point doses may serve as baseline for future comparison when evaluating the risk of optic neuropathy after SRS using different schedules.

The present study has some limitations. The small number of patients and the relatively short follow-up do not allow for definitive conclusions about the risk of symptomatic optic neuropathy when using multi-fraction SRS. Further studies and larger number of patients are needed to confirm that patients receiving doses >25 Gy to the optic nerve and chiasm had a low risk of developing clinically symptomatic radiation optic neuropathy. Nevertheless, our results suggest that when single doses to the anterior optic pathway exceeds 10–12 Gy, as for large skull base metastases or for those adjacent to the optic apparatus, multi-fraction SRS may represent an alternative to single-fraction SRS with similar local control and lower risk of treatment-related complications.

The 1-year and 2-year distant failure rates were 50% and 77%, necessitating salvage treatment with SRS in ten and WBRT in seven patients, respectively. Overall, WBRT could be avoided in about 75% of patients. The main justification for omitting WBRT is that it is associated with a decline in quality of life and neurocognitive function [10-13] without conferring survival advantages [12,13]. In a randomized study of 58 patients with 1 to 3 metastases who received WBRT plus SRS or SRS alone, Chang et al. [13] observed that patients treated with SRS plus WBRT showed a greater risk of a significant decline in learning and memory function at 4 months as compared with patients who received SRS alone. Sun et al. [11] reported similar neurocognitive impairment in patients with small cell lung carcinoma who received prophylactic WBRT. In our study the high distant failure rate was not apparently associated with increased death due to intracranial progression and neurological impairment, confirming that the omission of up-front WBRT is not detrimental for these patients. However, a close MRI follow-up is mandatory with the intent to treat early new distant brain metastases to avoid any potential neurocognitive deterioration.

In conclusion, multi-fraction SRS using 5 × 5 Gy is a feasible treatment option associated with good local control and improvement of cranial nerve symptoms in patients with a skull base metastasis involving the anterior visual pathway. A maximum dose less than 28.5 Gy delivered in 5 fractions to a small portion of the optic nerves and chiasm is associated with a very low risk of radiation-induced optic neuropathy.

## Competing interests

The authors declare that they have no competing interests.

## Authors’ contributions

GM conceived the study, participated in its design and coordination, and drafted the manuscript. VE, EC, CS, VDS, MME, and MV participated in study design, analysis and interpretation of data, and helped to draft the manuscript. EC and TF performed the statistical analysis and participated in acquisition and analysis of data. MFO and RME critically reviewed/revised the article. All authors read and approved the final manuscript.
